# F91. ASSOCIATION BETWEEN SYMPTOM DIMENSIONS AND EXECUTIVE FUNCTION IN SCHIZOPHRENIA

**DOI:** 10.1093/schbul/sby017.622

**Published:** 2018-04-01

**Authors:** Ana Luiza Franco, Bruno Bertolucci Ortiz, Felipe Ferreira, Ana Olívia Fonseca, Arthur de Almeida Berberian, Deyvis Rocha, Cristiano Noto, Rodrigo Bressan, Ary Gadelha

**Affiliations:** Universidade Federal de São Paulo (UNIFESP)

## Abstract

**Background:**

Impaired executive function is a core cognitive deficit in schizophrenia and strongly associated with functional outcomes. Understand the relationship between clinical symptoms and executive function may help the clinician to better manage the cognitive impairment and inform prognosis. The main objective of the present study was to investigate the association between symptom dimensions and executive function in schizophrenia.

**Methods:**

One-hundred and two patients with schizophrenia were recruited from the schizophrenia outpatient clinic from Universidade Federal de São Paulo (PROESQ/UNIFESP). Diagnosis was confirmed through the Structured Clinical Interview for DSM-IV (SCID-I) and dimensional psychopathology was assessed by the Positive and Negative Syndrome Scale (PANSS). The PANSS items were grouped in five factors: positive, negative, disorganized/cognitive, mood/depression and excitement/hostility factors. The cognitive battery included the following tests: Plus–Minus Task, Number–Letter Task, Trail Making Test - Part B, Keep Track Task, Letter Memory Task, Visual Working Memory Test – MTV, Stroop Test, Semantic Generation Task and The Tower of London Test – TOL. All tasks were computerized and assessed by the software Cronos. A single latent variable for executive function was derived through Confirmatory factor analysis and yield good model fits (CFI: 0.997; TLI: 0.996; RMSEA: 0.017; SRMR: 0.041).

**Results:**

When the factors were entered individually, negative (df=121, r=0,35, p <0.001) and disorganized (df= 121; r=-0.48, p <0.001) factors were significant predictors of EF. In a multivariate regression analysis, including all the factors and correcting for age, gender and duration of illness, only the disorganized factor remained significant (r^2^=0,21,p<0.001).

**Discussion:**

The disorganized factor was the symptomatic dimension more strongly associated with EF. The potential use of disorganized dimension as indicator of poor executive function and related outcomes, i.e., treatment resistant schizophrenia, should be further investigated.

